# Observing metabolic functions at the genome scale

**DOI:** 10.1186/gb-2007-8-6-r123

**Published:** 2007-06-26

**Authors:** Jean-Marc Schwartz, Claire Gaugain, Jose C Nacher, Antoine de Daruvar, Minoru Kanehisa

**Affiliations:** 1 Bioinformatics Center, Kyoto University, Uji, Kyoto 611-0011, Japan; 2 Faculty of Life Sciences, University of Manchester, Manchester M13 9PT, UK; 3 Centre de Bioinformatique de Bordeaux, Université Bordeaux 2, 33076 Bordeaux, France; 4 Department of Complex Systems, Future University, Hakodate, Hokkaido 041-8655, Japan

## Abstract

**Background:**

High-throughput techniques have multiplied the amount and the types of available biological data, and for the first time achieving a global comprehension of the physiology of biological cells has become an achievable goal. This aim requires the integration of large amounts of heterogeneous data at different scales. It is notably necessary to extend the traditional focus on genomic data towards a truly functional focus, where the activity of cells is described in terms of actual metabolic processes performing the functions necessary for cells to live.

**Results:**

In this work, we present a new approach for metabolic analysis that allows us to observe the transcriptional activity of metabolic functions at the genome scale. These functions are described in terms of elementary modes, which can be computed in a genome-scale model thanks to a modular approach. We exemplify this new perspective by presenting a detailed analysis of the transcriptional metabolic response of yeast cells to stress. The integration of elementary mode analysis with gene expression data allows us to identify a number of functionally induced or repressed metabolic processes in different stress conditions. The assembly of these elementary modes leads to the identification of specific metabolic backbones.

**Conclusion:**

This study opens a new framework for the cell-scale analysis of metabolism, where transcriptional activity can be analyzed in terms of whole processes instead of individual genes. We furthermore show that the set of active elementary modes exhibits a highly uneven organization, where most of them conduct specialized tasks while a smaller proportion performs multi-task functions and dominates the general stress response.

## Abstract 2

A modular approach is presented that allows the observation of the transcriptional activity of metabolic functions at the genome scale.

## Background

The increasing availability of high-throughput data has allowed more and more analyses to be performed at the cell scale. After completion of genome sequencing for many species, the focus is shifting towards getting a global understanding of cell physiology. This task requires the integration of heterogeneous data at different scales, including genomic, transcriptomic, proteomic, and metabolomic data.

At the level of metabolism, good knowledge of the structure of metabolic networks has now been achieved for several species. A number of genome-wide models of metabolism have been reconstructed [1-4], but these structural models provide only a static representation of an organism's metabolism; the structure of a metabolic network is static for a given species, and only changes at a slow pace across species through evolution [5]. However, the usage of particular metabolic reactions by a given cell is highly dynamic. It changes very rapidly in time with modifications in the environment, in the cell cycle, or with stochastic fluctuations. Static representations, therefore, need to be extended toward truly dynamic descriptions.

Metabolic networks are also highly complex, formed by several hundreds of densely interconnected chemical reactions. To characterize such complex systems at the genome scale, it is necessary to identify smaller building blocks. Cellular networks have been shown to have a high degree of modularity, and are composed of groups of interacting elements and molecules that carry out specific biological functions [6]. In recent years, several methods have been proposed to decompose complex biological networks into subnetworks and to identify basic interaction modules [5,7-9]. Although relevant progress has been achieved in detecting motifs and modules in transcriptional regulatory and protein-protein interaction networks [10-16], the building blocks of metabolic pathways still remain largely undiscovered. Evidence for the existence of modularity in metabolic pathways was recently proposed by Ravasz *et al*. [17], who showed that the high clustering degree observed in metabolic networks may imply a hierarchical modularity, in which modules are made up of smaller and denser modules in a fractal manner.

A complementary approach is provided by the concept of an 'elementary mode'. Elementary modes, and the very similar concept of 'extreme pathways', are minimal sets of reactions that can operate in steady state in a metabolic network [18-20]. They have already proven useful for studying many aspects of metabolism, including the prediction of functional properties of metabolic pathways, the measurement of robustness and flexibility, inferring the viability of mutants, the assessment of gene regulatory features, and so on [21]. Recently, it has been shown that they could even provide a basis for describing and understanding the properties of signaling and transcriptional regulatory networks [22,23]. All these applications, however, consider elementary modes as purely 'structural units'. Although the biological significance of elementary modes has already been mentioned [24], the use of elementary modes as true elementary 'functional units' of cellular metabolism has not been attempted so far. A few studies [25,26] have combined metabolic and transcriptomic data in order to find out whether co-expressed genes are part of a given metabolic pathway, but most of these approaches used complete metabolic pathways as metabolic units.

Here, we address the problem of identifying metabolic units in a genome-scale model of the yeast *Saccharomyces cerevisiae *by relying on elementary modes. Our study is based on the integration of dynamic gene expression data in various stress conditions into a genome-scale model of metabolism, modularly structured in elementary modes. We used a bioinformatics tool called BlastSets [27] to combine these two types of data in order to answer the following question: do enzymes that are involved in the same elementary mode have their corresponding genes co-expressed in particular conditions? We were able to identify active elementary modes, that is, elementary modes whose enzymes are induced or repressed in response to different environmental stresses; these elementary modes can thus be seen as functional units of the metabolic stress response.

## Results

### Genome-wide computation of elementary modes

The computation of elementary modes in genome-wide models of metabolism is seriously hampered by the problem of combinatorial explosion. Even though the number of elementary modes is usually smaller in a real system than its theoretical limit and can be further reduced by taking into account various environmental or regulatory constraints, it is of no practical use to handle systems of thousands of elementary modes because such systems become impossible to interpret [28,29]. One possible approach to deal with this problem consists of decomposing a genome-scale metabolic network into smaller subunits. This kind of decomposition has already been proposed, but was based on network topology [30]; it consisted of finding the optimal decomposition that minimized the number of elementary modes. However, there is no guarantee that such subunits represent functionally coherent and biologically interpretable pathways.

We have developed an alternative approach for computing elementary modes at the genome scale. In the Kyoto Encyclopedia of Genes and Genomes (KEGG) database, metabolic pathways are represented as a series of maps, where each map covers a precise biological function [31]. These maps are sufficiently small for the number of elementary modes inside each of them to remain in the hundreds (Table 1). Furthermore, because they have been manually drawn and annotated based on biological information, these units have a clear biological meaning and are easy to interpret. We thus considered each pathway map of the KEGG database as one subnetwork. We then computed the full set of elementary modes inside each of them using a classical algorithm [20] (Additional data file 1).

**Table 1:** KEGG metabolic pathways for *Saccharomyces cerevisiae *and number of elementary modes for each

Pathway identifier	Pathway name	Number of computed elementary modes	in BlastSets
sce00010	Glycolysis/gluconeogenesis	163	112
sce00020	Citrate cycle (TCA cycle)	99	60
sce00030	Pentose phosphate pathway	206	203
sce00040	Pentose and glucuronate interconversions	4	2
sce00051	Fructose and mannose metabolism	12	11
sce00052	Galactose metabolism	81	63
sce00053	Ascorbate and aldarate metabolism	2	2
sce00061	Fatty acid biosynthesis	4	3
sce00071	Fatty acid metabolism	22	20
sce00072	Synthesis and degradation of ketone bodies	4	2
sce00100	Biosynthesis of steroids	6	5
sce00120	Bile acid biosynthesis	5	4
sce00130	Ubiquinone biosynthesis	4	1
sce00190	Oxidative phosphorylation	7	7
sce00220	Urea cycle and metabolism of amino groups	12	11
sce00230	Purine metabolism	350	346
sce00240	Pyrimidine metabolism	31	28
sce00251	Glutamate metabolism	40	38
sce00252	Alanine and aspartate metabolism	43	39
sce00260	Glycine, serine and threonine metabolism	102	94
sce00271	Methionine metabolism	26	25
sce00272	Cysteine metabolism	14	12
sce00280	Valine, leucine and isoleucine degradation	8	7
sce00290	Valine, leucine and isoleucine biosynthesis	12	11
sce00300	Lysine biosynthesis	5	4
sce00310	Lysine degradation	6	5
sce00330	Arginine and proline metabolism	29	24
sce00340	Histidine metabolism	5	4
sce00350	Tyrosine metabolism	11	8
sce00360	Phenylalanine metabolism	3	3
sce00361	gamma-Hexachlorocyclohexane degradation	6	1
sce00362	Benzoate degradation via hydroxylation	3	0
sce00380	Tryptophan metabolism	15	8
sce00400	Phenylalanine, tyrosine and tryptophan biosynthesis	38	30
sce00401	Novobiocin biosynthesis	6	2
sce00410	beta-Alanine metabolism	6	6
sce00430	Taurine and hypotaurine metabolism	2	1
sce00440	Aminophosphonate metabolism	5	3
sce00450	Selenoamino acid metabolism	6	5
sce00460	Cyanoamino acid metabolism	9	2
sce00480	Glutathione metabolism	5	4
sce00500	Starch and sucrose metabolism	49	47
sce00520	Nucleotide sugars metabolism	15	11
sce00521	Streptomycin biosynthesis	2	1
sce00530	Aminosugars metabolism	13	13
sce00550	Peptidoglycan biosynthesis	3	0
sce00561	Glycerolipid metabolism	7	4
sce00562	Inositol phosphate metabolism	5	4
sce00563	Glycosylphosphatidylinositol (GPI)-anchor biosynthesis	3	0
sce00564	Glycerophospholipid metabolism	28	25
sce00590	Arachidonic acid metabolism	4	2
sce00600	Glycosphingolipid metabolism	7	5
sce00620	Pyruvate metabolism	139	132
sce00624	1- and 2-Methylnaphthalene degradation	7	3
sce00625	Tetrachloroethene degradation	4	1
sce00627	1,4-Dichlorobenzene degradation	9	0
sce00630	Glyoxylate and dicarboxylate metabolism	7	6
sce00632	Benzoate degradation via CoA ligation	7	2
sce00640	Propanoate metabolism	8	4
sce00650	Butanoate metabolism	9	7
sce00670	One carbon pool by folate	13	12
sce00680	Methane metabolism	5	3
sce00710	Carbon fixation	13	8
sce00720	Reductive carboxylate cycle (CO_2 _fixation)	3	3
sce00730	Thiamine metabolism	2	0
sce00740	Riboflavin metabolism	3	2
sce00750	Vitamin B6 metabolism	4	2
sce00760	Nicotinate and nicotinamide metabolism	9	8
sce00770	Pantothenate and CoA biosynthesis	4	3
sce00780	Biotin metabolism	1	1
sce00790	Folate biosynthesis	17	6
sce00860	Porphyrin and chlorophyll metabolism	4	3
sce00900	Terpenoid biosynthesis	9	8
sce00903	Limonene and pinene degradation	9	2
sce00910	Nitrogen metabolism	17	15
sce00920	Sulfur metabolism	3	2
sce00960	Alkaloid biosynthesis II	3	3
sce00970	Aminoacyl-tRNA biosynthesis	20	15
sce00980	Metabolism of xenobiotics by cytochrome P450	2	2
sce04070	Phosphatidylinositol signaling system	4	4

The first and second columns give the identifier and the name of each KEGG metabolic pathway. For each of them, the number of elementary modes computed is indicated in the third column and the number of elementary modes entered in the BlastSets database in the fourth column. In most cases, there is a difference between these two numbers because BlastSets eliminates redundant elementary modes and the ones involving only one enzyme.

**Figure 1:**
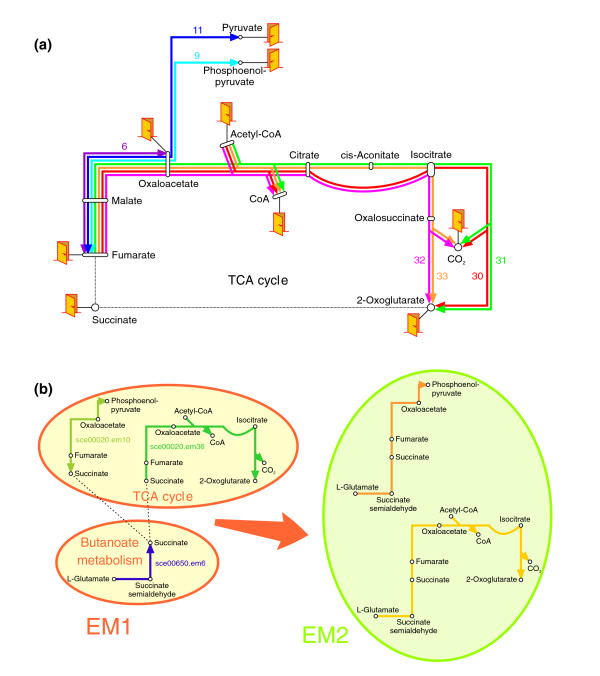
Construction of elementary mode collections. **(a) **This scheme represents some of the elementary modes calculated between fumarate and 2-oxoglutarate in the citrate cycle pathway. Each color corresponds to a different elementary mode; numbers indicate the identifiers of elementary modes as in Additional data file 1, and doors represent start and end compounds of elementary modes. This figure illustrates the combinatorial nature of elementary modes: several of them are almost identical except for one or two reactions, and a given reaction can belong to several elementary modes. **(b) **The composition of the EM1 collection (left) and how elementary modes were merged to build the EM2 collection (right). Three independent sets from EM1 can be merged into two sets in EM2 if they share a common boundary compound.

Because of their combinatorial nature, a number of different elementary modes usually share common reactions along their path. It often occurs that several elementary modes are almost identical except for a few branches at their extremities. Similarly, a given reaction can belong to a large number of different elementary modes. Figure 1a illustrates this property by showing some of the elementary modes between fumarate and 2-oxoglutarate in the citrate cycle (note that only 7 elementary modes have been drawn out of 99 calculated for the entire citrate cycle map). This combinatorial property, which is a major problem in large networks, is, on the contrary, welcome in our study: as our aim is to search for the most active route in a system, it guarantees that the full set of topologically possible routes will be considered in the search.

The use of KEGG maps for defining subnetworks aims at having entities that are as much as possible biologically coherent. The start and end points of elementary modes are compounds located at the boundaries between subnetworks. One drawback of this approach is that active metabolic routes that are spread over different KEGG maps may not be easily identified. To overcome this problem, we constructed two different collections of elementary modes, EM1 and EM2. EM1 contains the full set of single elementary modes computed with each KEGG pathway map being used as a subnetwork; each elementary mode from EM1 is entirely included in a single pathway map. EM2 was formed by combining all pairs of elementary modes from EM1 that are connected through a common boundary compound; elementary modes from EM2 thus spread over two different pathway maps (Figure 1b). The use of EM2 reduces the dependence of results on subnetwork boundaries since active elementary modes spread over different KEGG maps can now be identified. More details are provided in the 'Genome-wide computation of elementary modes' section in Materials and methods, and the full description of single elementary modes is available in Additional data file 1.

### Elementary modes represent true functional units of metabolism

#### Functional activity is more significant in elementary modes than in entire pathways

To elucidate whether elementary modes can be considered as true functional biological units, the stress response of yeast was investigated in a large number of different conditions. Towards this goal, we used microarray data obtained from several experimental analyses [32-34] (see the 'Expression data' section in Materials and methods) and a bioinformatics tool called BlastSets [27]. BlastSets enabled us to find similarities between the composition of two sets of genes or proteins derived from two different types of information (here, metabolic pathways and expression data). The elementary modes EM1 and EM2 were stored independently as two BlastSets collections. Entire KEGG pathways were also stored as a BlastSets collection, to find out whether stress responses involve entire pathways, as defined in KEGG, or only parts of these pathways, as represented by elementary modes. In many stress conditions, induced/repressed elementary modes were found with higher *P *values than whole pathways (Table 2).

**Table 2:** First induced/repressed pathway and first induced/repressed elementary mode in particular stress conditions

Stress condition	First pathway	*P *value	First elementary mode (EM1)	*P *value
Ash [34], repressed	sce00230 (purine metabolism)	2.7e-8	sce00230.em279 (part of purine metabolism)	1e-11
Pentanol [34], repressed	sce00230 (purine metabolism)	3.3e-6	sce00230.em341 (part of purine metabolism)	1.8e-8
Tetrachloro-isophthalonitrile [34], repressed	sce00230 (purine metabolism)	2.5e-8	sce00230.em280 (part of purine metabolism)	3.3e-10
Stationary phase [33], induced	sce00020 (citrate cycle)	3.4e-14	sce00020.em36 (part of citrate cycle)	5.9e-16
Heat shock [32], induced	sce00500 (starch and sucrose metabolism)	3.8e-4	sce00500.em13 (part of starch and sucrose metabolism)	4.2e-6

Results given by BlastSets for particular conditions. The second column gives the most significant full KEGG pathway found to be induced/repressed (that is, the one with the lowest *P *value, given in the third column). The fourth column gives the most significant elementary mode from EM1 found to be induced/repressed. These results are sorted from the highest to the lowest difference between the two *P *values.

The numbers of detected induced/repressed elementary modes for each stress condition are shown in Table 3, as well as the number of different KEGG pathways these elementary modes belong to. The numbers obtained with EM1 and EM2 are relatively well correlated but there is no absolute relationship between them; in most cases, the number of induced/repressed elementary modes is increased when compared to EM2, but a few of them show higher numbers with EM1. The same observation can be made about the number of KEGG pathways to which these elementary modes belong. In a majority of cases, elementary modes detected with EM1 are concentrated in a relatively small number of pathways, and EM2 increases this number by adding modes from adjacent pathways. But in a few cases, for example Thiuram, the number of pathways detected with EM2 is smaller than with EM1, indicating that these elementary modes tend to be isolated and poorly connected to adjacent pathways.

**Table 3:** Number of induced/repressed elementary modes in each condition

Stress condition	Number of induced or repressed elementary modes (EM1)	Number of induced or repressed KEGG pathways (EM1)	Number of induced or repressed elementary modes (EM2)	Number of induced or repressed KEGG pathways (EM2)
Heat shock [32], induced	12	2	28	4
Heat shock [32], repressed	2	2	2	2
NaCl [32], induced	5	1	4	2
Peroxide [32], induced	16	10	3	2
Sorbitol [32], induced	1	1	30	2
Acid [32], induced	6	1	0	0
Amino acid starvation [33], induced	13	3	104	19
Diamide [33], induced	42	12	196	21
Peroxide [33], induced	6	2	3	2
Heat shock [33], induced	34	2	88	7
Nitrogen depletion [33], induced	2	2	13	6
Stationary phase [33], induced	54	5	292	25
Variable temperature [33], induced	20	3	57	7
Ash [34], induced	24	11	153	19
Ash [34], repressed	200	2	284	8
Cadmium [34], induced	1	1	19	5
Maneb [34], induced	17	11	193	21
Octanol [34], induced	5	2	12	6
Pentachlorophenol [34], induced	7	5	56	12
Pentanol [34], induced	44	7	289	35
Pentanol [34], repressed	184	2	166	7
Thiuram [34], induced	12	11	19	5
Tetrachloro-isophthalonitrile [34], induced	17	11	25	8
Tetrachloro-isophthalonitrile [34], repressed	155	1	202	8
Zineb [34], induced	16	10	127	19

This table shows the number of elementary modes found induced or repressed in each stress condition. These include all the results given by BlastSets independently of their *P *value. The numbers given in the fourth column are the numbers of individual elementary modes and not the numbers of pairs.

**Figure 2:**
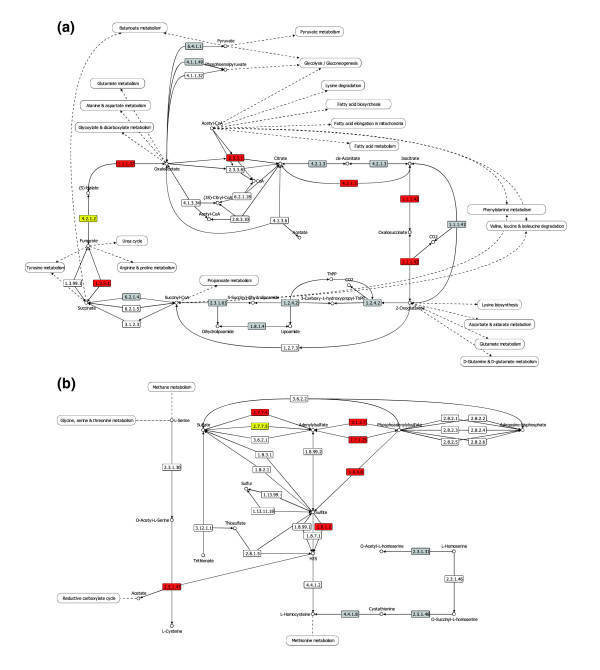
Examples of active elementary modes. **(a) **This figure shows the citrate cycle map from KEGG. Enzymes colored in red are coded by genes induced during the stationary phase. They correspond exactly to elementary mode number 36 of the citrate cycle, with the exception of one enzyme in yellow (4.2.1.2). **(b) **The sulfur metabolism map from KEGG. Enzymes colored in red are coded by genes found induced when yeast is exposed to tetrachloro-isophthalonitrile. These enzymes compose the entire elementary mode number 3 with the exception of two of them (in yellow): YGR012W is not induced but YLR303W is induced and fulfils the same function (EC 2.5.1.47); in the second case, two enzymes can fulfill the same function, so even if one is missing, the other completes the metabolic route (EC 2.7.7.5 and EC 2.7.7.4). Enzymes in grey are present in *S. cerevisiae *but do not belong to the elementary mode.

Examples of elementary modes induced in particular stress conditions are shown in Figure 2, including an induced elementary mode in the citrate cycle during stationary phase, and another induced one in sulfur metabolism in response to tetrachloro-isophthalonitrile exposure. The sets of induced enzymes detected by BlastSets are indeed highly connected. Fewer elementary modes could be identified from the sets of repressed enzymes and they are usually less connected, meaning that repressed enzymes are more dispersed in the mode. This fact has already been mentioned by Wei *et al*. [35] for the genetic model plant *Arabidopsis thaliana*, who observed that induced genes in the same metabolic pathway tend to be close and well connected to each other, while repressed genes are more distant.

#### Induced/repressed elementary modes are statistically significant

BlastSets applies a stringent threshold on *P *values (*P *value must be lower than 6.0 × 10^-5 ^for EM1 and 3.4 × 10^-6 ^for EM2; see 'Description of BlastSets' section in Materials and methods), which should already guarantee that identified elementary modes are statistically significant. Nevertheless, in order to further assess the reliability of our results, we created random gene expression values by random permutation of gene expression values in several stress responses. These random sets of induced/repressed genes were compared to elementary modes in BlastSets, in the same way as for stress-induced/repressed genes. No active elementary mode was identified using these random sets. The procedure was repeated for several conditions, always with the same result. This finding confirms that elementary modes found to be active in specific environmental stress conditions have a high statistical significance.

#### Pairing elementary modes to reconstruct induced/repressed routes

To identify complete metabolic routes that are spread over several KEGG pathway maps, we constructed the EM2 collection containing elementary modes grouped in pairs. Two elementary modes are grouped as a set in EM2 if they share a common boundary compound. These compounds act as bridges between individual pathway maps, enabling more extended induced/repressed routes to be identified by this approach.

In each stress situation, we could then infer a 'backbone' of induced/repressed metabolic routes. Backbones were constructed by selecting the pairs of elementary modes with the lowest *P *values and connecting them to each other, thanks to results from the EM2 collection (see 'Analysis of BlastSets results' section in Materials and methods). These backbones can be viewed as the main modules characterizing metabolic activity in terms of expression data in a given condition. They are provided for each individual condition in Additional data file 2.

#### Specialized and multitask elementary modes

**Figure 3:**
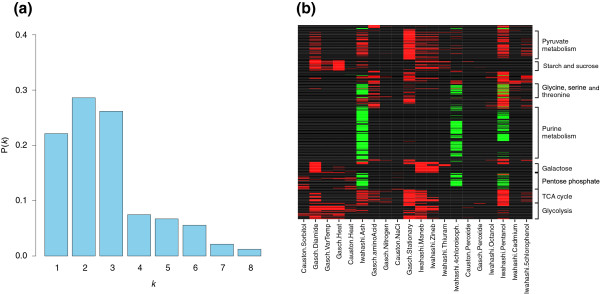
Transcriptional activity of elementary modes. **(a) **This histogram shows the probability of finding a given elementary mode induced/repressed in *k *stress conditions. **(b) **Map of genome-scale elementary mode activities. Each line of this figure corresponds to an elementary mode and each column to a stress condition. Repressed elementary modes are represented in green and induced modes in red.

To assess how the activity of elementary modes is distributed in response to a set of diverse environmental stresses, we computed the probability distribution *P*(*k*) to find a given induced/repressed elementary mode in *k *stress conditions (Figure 3a). This distribution reveals a highly heterogeneous behavior: on one hand, a relatively low number of 'multitask' elementary modes are transcriptionally active in a large number of different conditions, while on the other hand, many 'specialized' elementary modes are active in a small number of conditions (less than three). About 77% of detected elementary modes appear to be conducting specialized tasks while the remaining 23% are involved in the more general stress response. This observed metabolic organization is far from a random distribution, where each induced/repressed elementary mode would have the same chance to be active in the vicinity of the average value. The deviation from a random distribution suggests that elementary modes involved in the stress response are governed by a more complex organization [36], that is, that they are organized into complex modules across the metabolic network.

### Transcriptional activity of metabolic processes revealed by functional elementary modes

#### Map of elementary mode activities

It is possible to reveal the various patterns of stress responses by drawing the 'activity map' of elementary modes. In Figure 3b, each line represents an elementary mode and each column a stress condition; induced elementary modes are shown in red and repressed modes in green in this representation, which is deliberately chosen to look similar to a microarray. Indeed, in the same way a microarray represents a map of the transcriptional activity of individual genes, we are here able to construct a map of genome-scale elementary mode activities, revealing the transcriptional activity of entire metabolic processes. It is particularly clear on this map that most of the identified elementary modes are either only induced or only repressed. While the three repressed patterns are very similar, induced patterns are more diverse and very few elementary modes are induced over all conditions, confirming the trend revealed by the distribution in Figure 3a.

#### Two main classes of stress responses

Our approach is able to provide new insights about metabolic activity in terms of expression data in particular conditions. We analyzed the raw expression data obtained for each stress condition in order to see which stresses lead to similar responses; the clustering tree of stress conditions based on raw expression data is provided as Additional data file 3. Among the 31 different conditions we studied, 12 had a too weak transcriptional response for any induced or repressed elementary mode to be detected. We noticed that, among the remaining 19 conditions that produced a sufficiently strong response, stresses could be divided into two main classes, which we hence denote as 'toxic' and 'non-toxic'. The toxic stress class mostly includes exposure of cells to toxic chemicals and metals. The non-toxic class, on the contrary, mostly includes other types of stresses, such as temperature changes, osmotic shocks, nutrient starvation, and so on. The list of conditions assigned to each class is provided in Table 4.

**Table 4:** Composition of toxic and non-toxic stress classes

Toxic class	Non-toxic class	Not assigned
Peroxide [32]	Sorbitol [32]	Alkali [33]
Cadmium [34]	NaCl [32]	Dithiothreitol [33]
Maneb [34]	Acid [32]	Diauxic shift [33]
Octanol [34]	Heat shock [32]	Alternative carbon [33]
Pentachlorophenol [34]	Amino acid starvation [33]	Hypo-osmotic [33]
Pentanol [34]	Diamide [33]	Menadione [34]
Thiuram [34]	Nitrogen depletion [33]	n-Pentane [34]
Tetrachloro-isophthalonitrile [34]	Stationary phase [33]	Ethanol [34]
Zineb [34]	Variable temperature [33]	Sodium n-dodecyl benzosulfonate [34]
	Ash [34]	Sodium lauryl sulfate [34]
		Capsaicin [34]
		Trichlorophenol [34]

Composition of the toxic and non-toxic stress classes, determined from the clustering tree of stress responses. The third column contains conditions whose response was too weak for any elementary mode to be identified by BlastSets.

**Figure 4:**
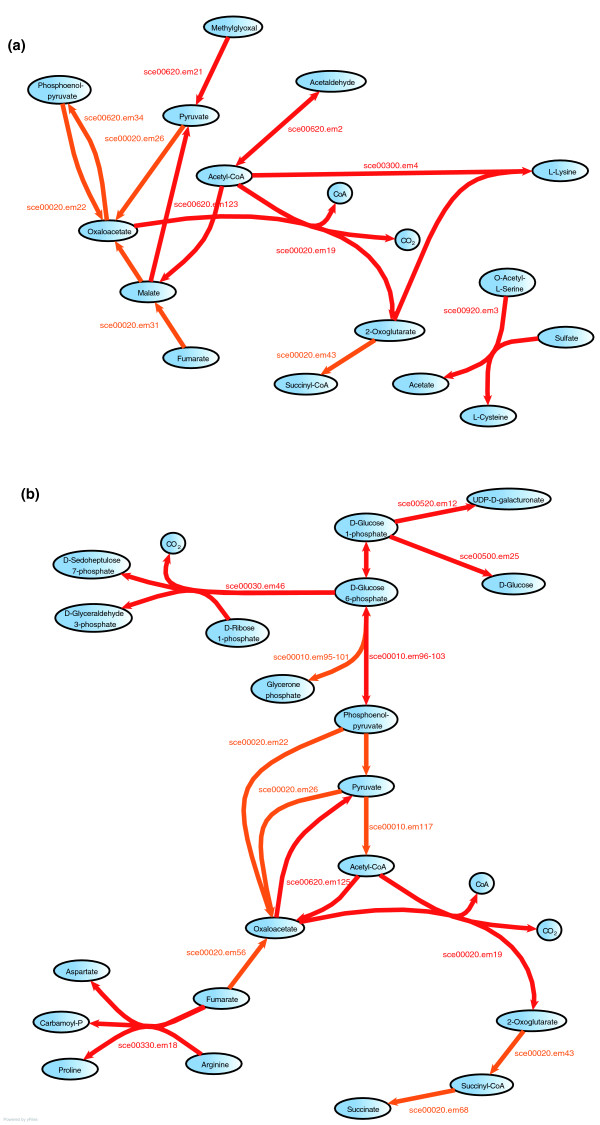
Backbones of metabolic stress response. **(a) **Toxic class. **(b) **Non-toxic class. These representations show all elementary modes induced in at least four different stress conditions. Main metabolic routes are drawn in red, and routes added by elementary modes that partly duplicate a main metabolic route but contain a separate short branch are drawn in orange.

**Figure 5:**
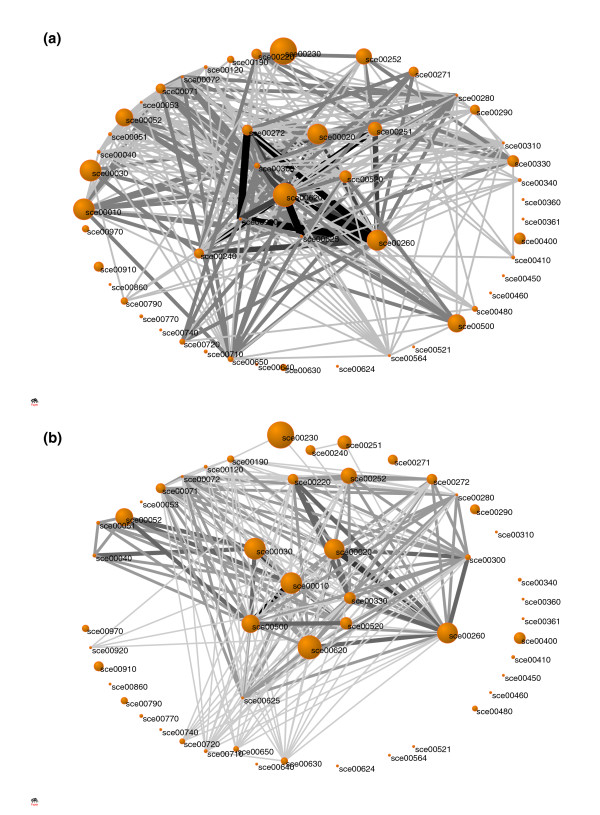
Interaction networks of metabolic pathways involved in the stress response according to the pairs of induced/repressed elementary modes spanning two pathways. **(a) **Toxic class. **(b) **Non-toxic class.

The metabolic backbones inside each class show recurrent similarities, which allowed us to construct a common backbone for each class (Figure 4). The two classes show a clearly distinct global response and few elementary modes are induced in both backbones, with the exception of the citrate cycle and nucleotide sugar metabolism. In addition, we represented both classes by networks where each node corresponds to a metabolic pathway and each edge denotes that at least one pair of elementary modes spanning both pathways is present in a stress response (see 'Construction of toxic and non-toxic networks' section in Materials and methods). The toxic response network is shown in Figure 5a and exhibits two components. The inner component is composed of a group of strongly connected pathways centered on sulfur metabolism, pyruvate metabolism and lysine biosynthesis metabolism. These pathways thus have a strong tendency to be activated simultaneously. They constitute the core of the toxic stress response and cover most parts of the toxic backbone described previously. The external component, in contrast, is composed of a sparse network with thinner connections. In the non-toxic network this bi-component nature is less clear, but it is still possible to identify a more strongly connected central component containing starch and sucrose metabolism, the pentose phosphate pathway, glycolysis, and arginine and proline metabolism (Figure 5b).

#### Insights about specific stress conditions

In some cases, the observed transcriptional metabolic response confirms earlier findings. Vido *et al*. [37] reported that cadmium exposure increases the synthesis of cysteine and perhaps of glutathione, which is essential for cellular detoxification. The synthesis of these two compounds is possible through the activation of the sulfur amino acid pathway. We observe that, among the three elementary modes activated in response to cadmium exposure, two have cysteine as their final product, and among these two, one elementary mode is a part of cysteine metabolism and another is a part of sulfur metabolism. Cysteine is also one of the compounds produced in the general backbone of the response to toxic stresses (Figure 4a).

Amino acid starvation is known to activate the transcription factor Gcn4p, which induces genes involved in amino acid biosynthetic pathways, except the cysteine pathway [38], although the genes involved in the biosynthesis of cysteine precursors (homocysteine and serine) are induced. This is exactly what we observe in response to amino acid starvation: several elementary modes from amino acid biosynthetic pathways are activated but none from the cysteine pathway, even if some elementary modes from the cysteine pathway are linked to modes activated during amino acid starvation.

Genes induced in stationary-phase cultures of yeast are associated with mitochondrial functions, that is, aerobic respiration and the citrate cycle [39]. ATP synthesis is thus very important for yeast in the stationary phase. In our results, the elementary modes activated during the stationary phase are part of metabolic pathways linked to aerobic respiration, including glycolysis, the citrate cycle, pyruvate metabolism and oxidative phosphorylation.

Trehalose and glycerol are produced in large amounts by cells in stress situations [40]. Schade *et al*. [40] have shown that there is an overlap between the late cold response and the environmental stress response. This response corresponds to the production of glycerol and trehalose. This is what we observed in the general non-toxic backbone response (Figure 4b): glycerol is produced just a few reactions after glycerone phosphate, and trehalose is present one step before D-glucose in the starch and sucrose metabolism KEGG map (the only reason why it cannot appear as an end product in our study is that it is not a boundary compound in KEGG maps). These examples, confirming previously observed results, enable us to be confident in the identification of metabolic processes found to be induced/repressed in response to other stress conditions.

## Discussion

There have been growing developments in recent years towards a more systems-level approach for understanding living organisms. On one side, microarray technologies have generalized the study of the transcriptome of biological cells in various conditions, and on the other side, numerous efforts have been undertaken to construct and describe the properties of metabolic networks at the genome scale. It is timely, therefore, to integrate both efforts and move towards a genome-scale analysis of cell metabolism.

At the same time, it is believed that a better understanding of the metabolome will be an important step towards improving the efficiency of the drug discovery process [41]. Instead of concentrating on the 'genomic universe', that is, the levels of gene regulation and transcription, our approach shifts the focus to the 'biochemical universe', that is, the small molecules or metabolites that actually perform biological functions and allow organisms to live and thrive. This shift is symbolized by the microarray-style representation of Figure 3b, which instead of showing the transcriptomic activities of individual genes, displays the transcriptomic activity of entire metabolic functions, represented by elementary modes, at the genome-scale. Although this is still a long way from an accurate and quantitative representation of the actual metabolic activity of a whole cell, which would require metabolic flux measurements, we believe that this shift opens a new perspective with a wide range of potential applications.

A major challenge addressed in this work consisted of embedding a suitable modularity into the highly complex and interconnected structure of metabolic networks. Our approach for computing elementary modes at the genome scale using KEGG pathway maps presents a number of advantages. These maps provide a decomposition of the metabolic network into well-defined subnetworks, which are biologically coherent and easy to interpret. Each map is sufficiently small for the number of elementary modes to remain in the hundreds, thus avoiding the necessity of having to cope with the problem of combinatorial explosion of elementary modes in large systems. Furthermore, these maps provide a manually curated representation of metabolic pathways where most secondary metabolites have been removed, thus avoiding the need to use complex procedures to identify principal metabolic routes and to eliminate invalid metabolic connections.

Microarray experiments are subject to a number of factors and we observed discrepancies in data obtained by different authors in similar conditions (peroxide treatment and heat shock experiments are available from both Gasch *et al*. [33] and Causton *et al*. [32]) The question of reproducibility of microarray experiments has been recurrent, but large-scale cross-platform experiments have shown that microarray data are indeed reliable and reproducible when adequate care is taken in experimental design and data treatment [42]. Differences may indicate that the transcription of genes involved in the metabolic response to stress is finely regulated and can fluctuate depending on a large number of factors.

Challenges also remain to obtain a more accurate description of the transcriptional activity of elementary modes in a cell. Our approach can be seen as 'discrete', since an elementary mode can only be assigned to three possible categories, that is, induced, repressed, or inactive. This division into three categories relies on a threshold on expression fold-change values, but enhanced statistical approaches could be researched to obtain a more subtle classification and avoid the need to set a threshold. Furthermore, with BlastSets, the localization of induced/repressed genes 'inside' an elementary mode is not taken into account, although this information could be relevant. For example, a repressed gene belonging to a group of genes coding for the same enzyme may have no influence on the activity of the elementary mode as a whole, while repression of a gene that is the only one to encode a particular enzyme would be important. Finally, the computation of elementary modes is based on a steady-state assumption and it remains to be seen to what extent these concepts can be extended to dynamic activity.

## Materials and methods

### Genome-wide computation of elementary modes

Combinatorial explosion prevents the computation of elementary modes in large networks. For example, in a network of about 110 reactions the number of elementary modes was shown to be higher than two million [21]. Furthermore, even if more efficient algorithms were found, it would not be particularly useful to compute all the elementary modes in a genome-wide model of metabolism because the resulting set would be extremely difficult to interpret. We therefore opted for an alternative modular approach for computing elementary modes at the genome scale. Pathway maps of the KEGG database constitute a good basis for this task, as each of them represents a coherent and well-defined biological function and is sufficiently small for the number of elementary modes to remain in the hundreds.

We used the KEGG XML files for *S. cerevisiae *as a source for the metabolic model [43]. These files have the advantage of having been manually curated and they contain the same information as the graphical maps displayed by the KEGG database. They thus have been cleaned from invalid metabolic connections due to very common compounds (ADP, ATP, and so on), which otherwise create artificial links between metabolic compounds that do not correspond to biologically valid metabolic routes.

A stoichiometric matrix was constructed for each pathway based on its XML description. A point of major importance to the computation of elementary modes is the definition of 'external metabolites'. They act as start and end points of elementary modes, and in our hierarchical approach they additionally enable elementary modes from different pathway maps to be connected to each other. We adopted the following rules for defining external metabolites: one, a metabolite located at the interface between two or more pathway maps is considered external to all of them; two, a metabolite that can only be either produced or consumed is considered external; and three, unbalanced ubiquitous metabolites are considered external. Rule one creates the vast majority of entry/exit points to elementary modes and allows connections between pathway maps. Rule two prevents the existence of 'inactive' metabolic branches, that is, branches of a metabolic network that cannot participate in any elementary mode. This happens, for example, when a branch ends up in a dead end: as steady state conditions are assumed when elementary modes are computed, no flux can be present in a dead-end branch since this would lead to accumulation of the compound at its extremity. Rule three was introduced to prevent particular branches of the metabolic network from collapsing due to inappropriate balancing. For example, CO_2 _appears on the map of the citrate cycle and must be considered external for the cycle to be able to operate, otherwise this route would contain a dead end and become inactive for the reason stated above. The complete list of metabolites covered by rule three comprises H_2_O, O_2_, P, CoA, CO_2_, NH_3_, UDP, H_2_, and reduced and oxidized thioredoxin.

Once stoichiometric matrices had been constructed, elementary modes were computed using a classical algorithm [20]. The complete list of elementary modes for *S. cerevisiae *is provided as Additional data file 1, and was used to create the EM1 and EM2 BlastSets collections.

### Expression data

#### Data sources

We have chosen experiments analyzing the gene expression responses of the yeast *S. cerevisiae *to various environmental stresses. Three sets of microarray experiments have been selected for our study. Causton *et al*. [32] described the transcriptional response to environmental changes using genome-wide expression experiments; data are available on the Young lab website [44]. Gasch *et al*. [33] analyzed gene expression of yeast cells during the adaptation to stressful environments in order to identify the main patterns of response in these different conditions; data were downloaded from the Stanford MicroArray Database website [45]. Iwahashi *et al*. [34] studied transcriptional responses of yeast to physical and chemical stresses using microarray; data are available from the Yeast Environmental Stress database [34].

These three datasets enabled us to study a total of 31 stress conditions. Some of these stresses involved environmental changes or nutrient depletion, while others involved exposure to toxic compounds such as pesticides or fungicides. The latter include: ash, which refers to exposure to burned ash from an industrial incinerator; maneb, which is a fungicide used in the control of several diseases of fruit, vegetable, field crops and ornamentals; pentachlorophenol (PCP), which is an effective fungicide, herbicide and algicide used as a wood preservative; tetrachloro-isophthalonitrile (TPN), which is a fungicide used to prevent biofouling on ships and in agriculture; thiuram, which is a compound used as fungicide to prevent crop damage and to protect harvested crops; and zineb, which was rated as a pesticide of low toxicity and may be a weak mutagen.

#### Data processing

We plotted the distributions of the natural logarithm of fold-change values for the Causton, Gasch and Iwahashi datasets. For each of the three sets of data, the standard deviation was determined. A threshold was defined by multiplying the standard deviation by a constant, and this threshold was used to determine which genes were considered as significantly induced or repressed in each condition. Genes whose fold change was higher than the threshold were considered induced; genes whose fold change was lower than 1 divided by the threshold were considered repressed. For each condition, a set of induced genes and a set of repressed genes were constructed. Table 5 indicates the number of genes present in each set.

**Table 5:** Number of genes in each induced and repressed set

Stress condition	Number of genes in induced set (in BlastSets)	Number of genes in repressed set (in BlastSets)
Heat shock [32]	173	2
Acid [32]	32	6
Alkali [32]	73	10
Peroxide [32]	99	35
NaCl [32]	193	113
Sorbitol [32]	136	8
Heat shock [33]	114	0
Nitrogen depletion [33]	167	11
Stationary phase [33]	334	0
Hyperosmotic [33]	20	0
Peroxide [33]	60	0
Diauxic shift [33]	17	0
Menadione [33]	30	14
Dithiothreitol [33]	56	0
Hypoosmotic [33]	11	0
Diamide [33]	94	0
Variable temperature [33]	91	0
Amino acid starvation [33]	61	7
Alternative carbon [33]	0	0
Cadmium [34]	149	16
Ash [34]	390	713
Sodium n-dodecyl benzosulfonate [34]	36	2
Sodium lauryl sulfate [34]	53	2
Capsaicin [34]	10	0
Thiuram [34]	273	166
Zineb [34]	62	17
Maneb [34]	21	4
Tetrachloro-isophthalonitrile [34]	347	518
Pentachlorophenol [34]	181	31
Trichlorophenol [34]	27	10
Ethanol [34]	210	30
Pentanol [34]	285	182
Irradiation [34]	6	6
Octanol [34]	119	55
Pentane [34]	28	40

The number of genes identified by BlastSets in each stress condition from the three sets of microarray experiments.

#### Creation of random data sets

For three particular conditions (one from each dataset), we re-assigned gene expression values randomly to all genes of the experiment. We then processed these random expression data using the same procedures as described above. The resulting sets of random induced/repressed genes were compared to elementary modes using BlastSets in the same way as real expression data.

### Data integration and analysis

#### Description of BlastSets

BlastSets is a bioinformatics tool that enables the integration of various biological data. This tool uses a standard representation for all types of data: data are structured in collections of sets of genes or proteins. Each collection corresponds to a biological source of information, and sets are composed of genes that share a similar property or value (close genes on a chromosome, co-expressed genes, proteins belonging to the same complex, proteins involved in the same metabolic pathway, and so on). The sets stored in the BlastSets database can be compared to each other or to submitted custom sets. To evaluate the similarity between two sets, their composition in terms of genes/proteins is compared, and the hypergeometric distribution is used to decide if the number of genes in common between the two compared sets is statistically significant (*P *value). As an example, one can check if the genes found co-expressed in an experiment correspond to a set containing proteins involved in the same pathway.

A *P *value is considered significant by BlastSets if it is less than or equal to a certain threshold. Multiple comparisons are performed as a set is compared to a collection of sets. The *P *value significance threshold is thus adjusted to the considered target sets using a Bonferroni correction. This takes into account the number of comparisons conducted, which depends on the number of sets in the collection. The resulting threshold is 6.0 × 10^-5 ^when a set is compared to EM1 and 3.4 × 10^-6 ^when compared to EM2. All hits with higher *P *values were automatically rejected. Additional details about BlastSets can be found in [27].

#### Integration of elementary modes in BlastSets

We used BlastSets to evaluate the biological relevance of elementary modes by comparing them to the sets of induced or repressed genes described above. We created two different collections of sets of elementary modes named KEGG_EM_1 (EM1) and KEGG_EM_2 (EM2) in BlastSets. EM1 is a collection of single elementary modes, that is, enzymes involved in a given elementary mode are gathered in a set labeled by the name of the mode. EM2 is a collection of pairs of elementary modes: all enzymes involved in two elementary modes that are connected through a common external link form one set in EM2. These two collections of sets of elementary modes are stored in the BlastSets database, and can be queried against user-submitted data via the BlastSets website [46].

#### Analysis of BlastSets results

Sets of induced and repressed genes in various stress conditions were compared to elementary modes using BlastSets, and lists of elementary modes found to be similar to the submitted sets of induced/repressed genes were obtained. A Perl script was developed to analyze these results and, thus, make it possible to reconstruct the chain of elementary modes that have been activated or repressed in response to each stress condition.

We retrieved the elementary modes (single or pair) that had the highest similarity with the set of induced/repressed genes, called the 'best hit'. First, if the best hit was a single elementary mode, we browsed subsequent hits until we found a pair of elementary modes containing this best hit. Second, once this 'best elementary mode pair' had been found, the rest of the list was browsed in order to find further pairs of elementary modes that were connected to the best pair, that is, pairs of elementary modes having one mode in common with the best elementary mode pair. Third, we could display a chain of pairs of elementary modes that defines the backbone of the metabolic response. If the best hit was a pair of elementary modes, only the second and third steps were performed. Among the elementary modes that could be added to the backbone, we removed all those that were composed of less than three enzymes to ensure that they were significant enough and to avoid the inclusion of short modes that are not specific to a single pathway.

#### Construction of toxic and non-toxic networks

Using the files containing BlastSets results with EM2, we constructed a matrix representing the usage of elementary modes in response to the different stresses, each row corresponding to an elementary mode and each column corresponding to a stress condition. In each element of the matrix, 1 was entered if the elementary mode was identified in response to the stress, 0 if it was not. A program was developed to compute, for each pair of pathways, the number of conditions where at least one pair of elementary modes spanning both pathways was found to be induced.

In Figure 5, each pathway was represented by a node whose radius was set proportional to the natural logarithm of the number of elementary modes contained in that pathway (for pathways with only one mode, the value was set to 0.5). This radius does not depend on the stress response and is only aimed at enhancing large pathways. Two pathways were connected by an edge if the number of induced pairs of elementary modes spanning both of them was non-zero. We weighted the connections by setting the thickness of edges proportional to the number of stress conditions in which such pairs were found. The weight thus does not depend on the number of active elementary modes in both pathways but on the number of conditions where both pathways contain simultaneously activated elementary modes. For a clearer representation, all weights were reduced by one unit, so that edges of weight 1 are not visible and the smallest visible edges are those of weight 2.

## Additional data files

The following additional data are available with the online version of this paper. Additional data file is a list of elementary modes for *Saccharomyces cerevisiae*. Additional data file 2 is a figure showing induced and repressed metabolic backbones for all stress conditions. Additional data file 3 is a figure of a clustering tree of stress conditions.

## Supplementary Material

Additional data file 1 Elementary modes for *Saccharomyces cerevisiae*. Click here for file

Additional data file 2 Induced and repressed metabolic backbones for all stress conditions. Click here for file

Additional data file 3 Clustering tree of stress conditions. Click here for file
