# Supplementary figures and images for: Observing metabolic functions at the genome scale
Source: Genome Biol. 2007 Jun 26;8(6):R123. doi: 10.1186/gb-2007-8-6-r123 (PMC2394767; doi:10.1186/gb-2007-8-6-r123)

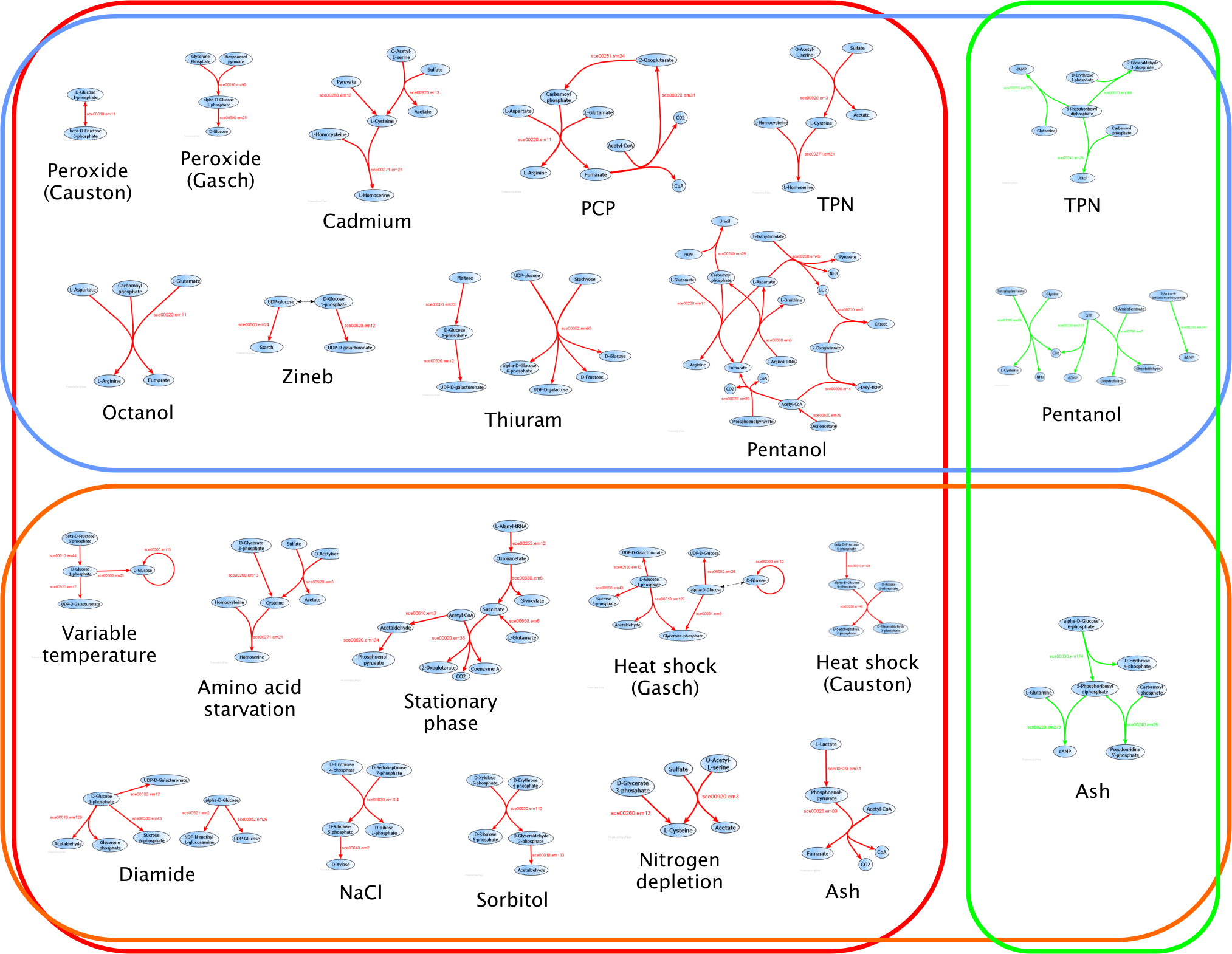

Supplement: Additional data file 2 — Induced and repressed metabolic backbones for all stress conditions. [file gb-2007-8-6-r123-S2.tiff]

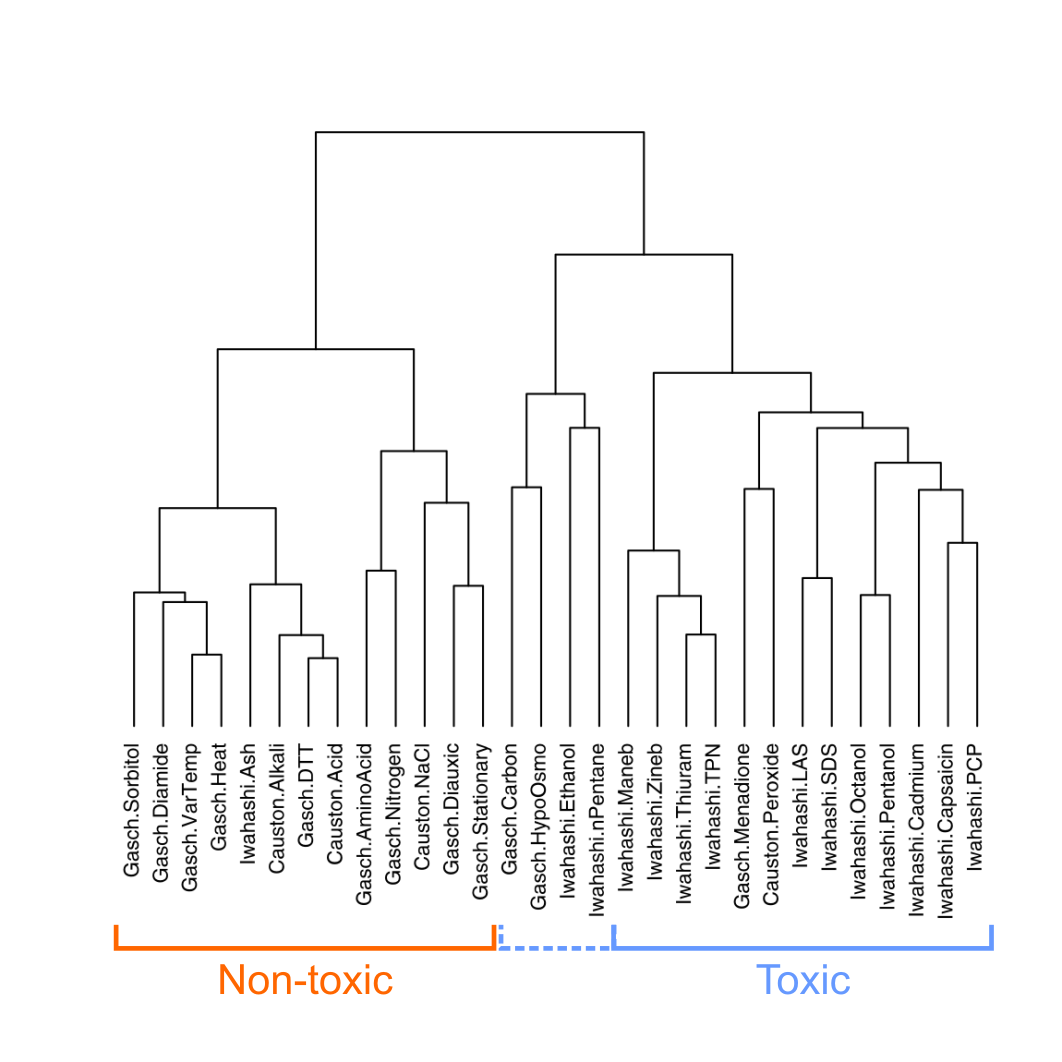

Supplement: Additional data file 3 — Clustering tree of stress conditions. [file gb-2007-8-6-r123-S3.tiff]
